# Development and Validation of a Prognostic Model for Post-Operative Recurrence of Pituitary Adenomas

**DOI:** 10.3389/fonc.2022.882049

**Published:** 2022-04-28

**Authors:** Liang Lu, Xueyan Wan, Yu Xu, Juan Chen, Kai Shu, Ting Lei

**Affiliations:** Department of Neurosurgery, Tongji Hospital, Tongji Medical College, Huazhong University of Science and Technology, Wuhan, China

**Keywords:** pituitary adenoma, tumor recurrence, nomogram, pseudocapsule, extracapsular resection, cavernous sinus invasion

## Abstract

**Background:**

We aimed to assess clinical factors associated with tumor recurrence and build a nomogram based on identified risk factors to predict postoperative recurrence in patients with pituitary adenomas (PAs) who underwent gross-total resection (GTR).

**Methods:**

A total of 829 patients with PAs who achieved GTR at Tongji Hospital between January 2013 and December 2018 were included in this retrospective study. The median follow-up time was 66.7 months (range: 15.6–106.3 months). Patients were randomly divided into training (n = 553) or validation (n = 276) cohorts. A range of clinical characteristics, radiological findings, and laboratory data were collected. Uni- and multivariate Cox regression analyses were applied to determine the potential risk factors for PA recurrence. A nomogram model was built from the identified factors to predict recurrence. Concordance index (C-index), calibration curve, and receiver operating characteristic (ROC) were used to determine the predictive accuracy of the nomogram. Decision curve analysis (DCA) was performed to evaluate the clinical efficacy of the nomogram.

**Results:**

Pseudocapsule-based extracapsular resection (ER), cavernous sinus invasion (CSI), and tumor size were included in the nomogram. C-indices of the nomogram were 0.776 (95% confidence interval [CI]: 0.747–0.806) and 0.714 (95% CI: 0.681–0.747) for the training and validation cohorts, respectively. The area under the curve (AUC) of the nomogram was 0.770, 0.774, and 0.818 for 4-, 6-, 8-year progression-free survival (PFS) probabilities in the training cohort, respectively, and 0.739, 0.715 and 0.740 for 4-, 6-, 8-year PFS probabilities in the validation cohort, respectively. Calibration curves were well-fitted in both training and validation cohorts. DCA revealed that the nomogram model improved the prediction of PFS in both cohorts.

**Conclusions:**

Pseudocapsule-based ER, CSI, and tumor size were identified as independent predictors of PA recurrence. In the present study, we developed a novel and valid nomogram with potential utility as a tool for predicting postoperative PA recurrence. The use of the nonogram model can facilitate the tailoring of counseling to meet the individual needs of patients.

## Introduction

Pituitary adenomas (PAs), which represent 10–20% of all brain tumors, are benign tumors with a prevalence rate of 80–100/100,000 (1, 2). Further, the incidence of clinically related PAs is 4–7/100,000 per year (3). The prevalence and the incidence of PA are increasing due to the increased availability of MRI (4). Autopsy and radiological studies demonstrated that PAs have a prevalence rate of 17% (range: 14–23%), which is higher than expected (5). The primary treatment for PAs is transnasal-transsphenoidal surgery (TTS) (6); however, suprasellar or parasellar PAs are difficult to remove completely and result in residual adenoma relapse in 12–58% of patients (1). Even in adenomas achieving GTR, 10–20% recur within 5–10 years (1, 7). In patients who undergo repeated treatment for recurrent PAs, mortality rates are elevated and quality of life is significantly affected due to pituitary dysfunction, invasion-related risks, and complications (8). Therefore, the identification of predictive factors for recurrent PAs is needed.

A pseudocapsule is defined as the boundary between the pituitary gland and an adenoma, which results from compressed peritumoral cell cord basement membrane condensation (9). PAs often spread beyond the edge of the pseudocapsule and invade the surrounding pituitary tissue (10). These characteristics of PAs have resulted in an alteration of their operative approach including extending excision boundaries to achieve total tissue removal and providing special attention to complete pseudocapsule removal (11). Although some studies have reported that pseudocapsule-based ER may improve the resection rate and reduce the recurrence rate (11, 12), there have been few reports on the relationship between pseudocapsule-based ER and PA recurrence after GTR.

A nomogram is a convenient graphical representation of a model that includes various significant factors to predict a specific outcome. Two nomograms related to PA recurrence have been constructed for non-functional PAs and giant PAs, respectively (13, 14). Good performance has been verified in the two types of PAs in the above two nomograms, in which PA recurrence included regrowth of tumor remnants after subtotal resection or partial resection. In the present study, a nomogram was constructed and validated to predict all subtypes PA recurrence after GTR by combining clinical variables including pseudocapsule-based ER and clinical image data.

## Materials and Methods

### Study Population and Design

**Figure 1:**
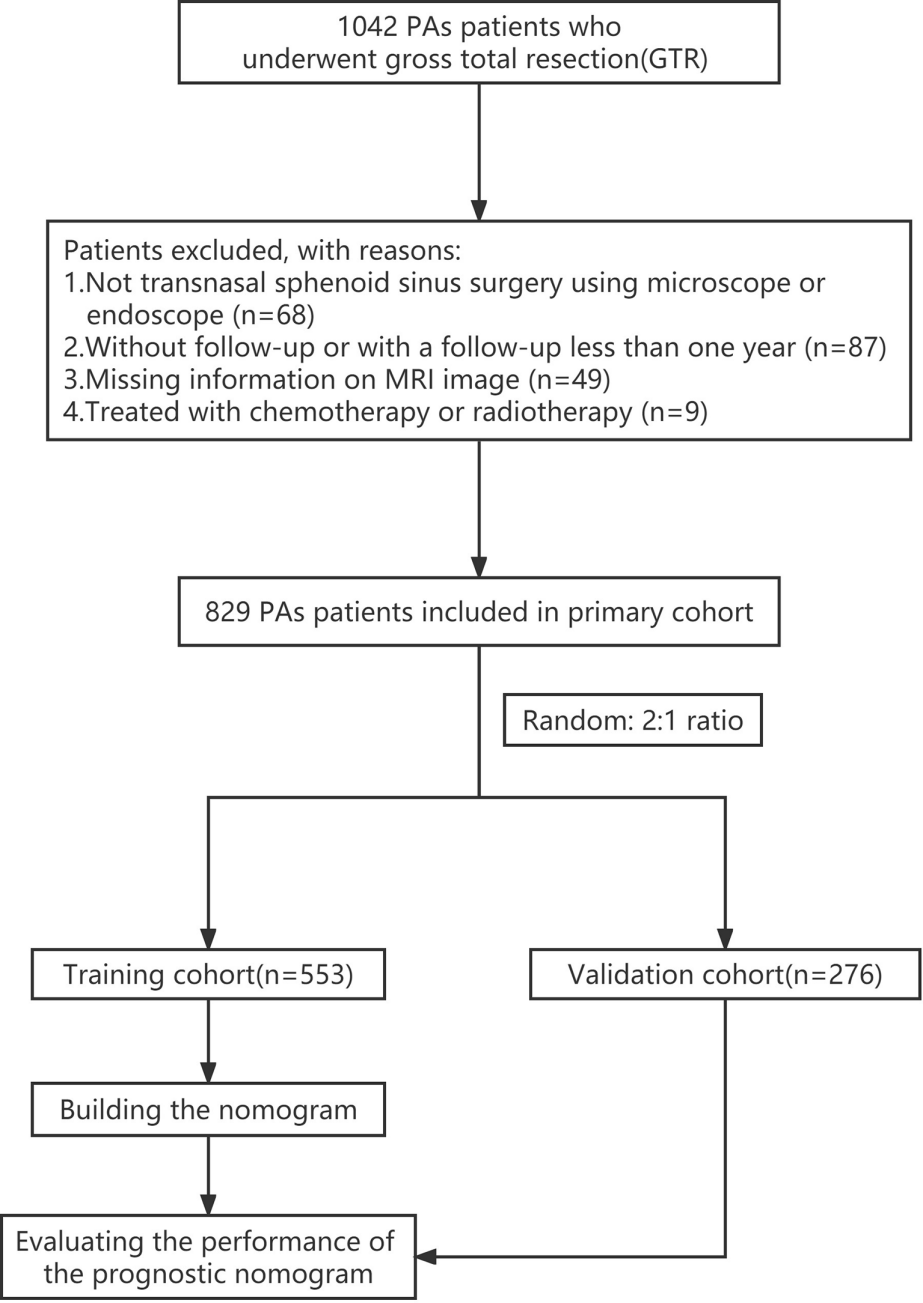
Flowchart summarizing the enrollment strategy and design of the study. PAs, pituitary adenomas.

We retrospectively assessed patients with PAs who underwent TTS at Tongji Hospital between January 2013 and December 2018. The median follow-up time was 66.7 months (range: 15.6–106.3 months). The criteria for inclusion were as follows: (1) PA patients confirmed histologically; (2) patients who underwent microscopic or endoscopic TTS; (3) patients who achieved GTR; and (4) patients followed up for more than one year post-GTR. Criteria for exclusion were as follows: (1) patients who did not undergo follow-up evaluations; (2) patients treated with chemotherapy or radiotherapy; and (3) patients with no data relating to variables assessed. The primary cohort was randomly assigned to the training and internal validation cohorts at a ratio of 2:1, respectively. A flowchart summarizing the enrollment strategy and design of the study is shown in **Figure 1**.

The following information was collected from all enrolled patients: baseline characteristics, radiological features, and preoperative laboratory tests. PA recurrence was defined as the reappearance of PAs, as observed *via* MRI examination after GTR. CSI status was determined according to MRI and operative records within which operating neurosurgeons documented their impressions. Pseudocapsule-based ER was conducted on patients with PAs within whom a pseudocapsule was identified during surgery. PFS in patients with PAs measured from the time of TTS to tumor recurrence. The study was approved by the Ethics Committee of Tongji Hospital. Due to the retrospective nature of the cohort study, the need for informed consent was waived.

### Development and Validation of the Nomogram

There were no missing data among clinical characteristics. For radiological findings and laboratory data, mean imputation for missing data was applied. Patients were randomly assigned to the training and validation cohorts. Based on the rule of having at least 10 outcome events per variable (EPV) (15), we ensured no more than six features were retained for multivariate Cox regression analysis from 63 events in the training cohort.

All factors were filtered by least absolute shrinkage and selection operator (LASSO) algorithm using the R package glmnet (version 4.1). Uni- and multivariate Cox regressions were utilised to confirm independent risk factors related to recurrence level using the R package rms (version 6.2). Variables included in the multivariate Cox regression analysis were required to meet at least one criterion: confirmation as a significant predictor in comparison of recurrence and non-recurrence cohorts or univariate Cox regression analysis, or retention in LASSO analysis. Using the R packages rms and survival (version 3.2), identified risk factors were used to develop a nomogram. C-index, ROC curve analysis, and calibration curves were used to evaluate the accuracy of the nomogram model, and DCA was used to evaluate the clinical utility of the model.

### Statistical Analysis

Model construction and validation were carried out on the basis of “Transparent Reporting of a Multivariable Prediction Model for Individual Prognosis or Diagnosis” (TRIPOD) guidance (**Table S1**) (15). R software (version 3.6.3) was used to perform statistical analysis and p < 0.05 was considered statistically significant. Categorical data were presented as percentages and continuous variables as means ± standard deviation (SD). The Student’s t-test was used to compare two continuous variables, and the Chi-square test or Fisher’s exact tests were used for categorised variables. The Spearman’s correlation test was used to verify correlations between the quantitative variables and the Kaplan–Meier method was used for estimating PFS. Data were visualised using the “ggplot” R packages (version 3.3.3).

## Results

### Clinical Characteristics

Detailed characteristics of patients with PAs in the recurrence and non-recurrence cohorts are summarized in **Table S2**, and the baseline characteristics of PA patients in the training and validation cohorts are summarised in **Table 1**. The training cohort included 315 male patients and 238 female patients, with an average age of 49.8 ± 12.36 years and a mean tumor size of 23.4 ± 5.5 mm. The validation cohort included 153 males and 123 females, with an average age of 49.5 ± 12.45 years and a mean tumor size of 23.7 ± 5.9 mm. Recurrence rates of the training and verification cohorts were 11.4% and 11.2%, respectively. No statistically significant between-cohort differences in patient characteristics were observed.

**Table 1:** Characteristics of patients with PAs in the training and validation cohorts.

Variable	Training cohort (n=553)	Validation cohort (n=276)	P value
Recurrence			0.945
YES	63 (11.4%)	31 (11.2%)	
NO	490 (88.6%)	245 (88.8%)	
Age, year	49.8 ± 12.36	49.5 ± 12.45	0.897
Gender			0.676
Female	238 (43.0%)	123 (44.6%)	
Male	315 (57.0%)	153 (55.4%)	
Headache			0.441
YES	148 (26.8%)	67 (24.3%)	
NO	405 (73.2%)	209(75.7%)	
Visual impairment			0.291
YES	273 (49.4%)	147 (53.3%)	
NO	280 (50.6%)	129(46.7%)	
Visual field defect			0.870
YES	125 (22.6%)	61 (22.1%)	
NO	428(77.4%)	215 (77.9%)	
Abnormal Menstruation			0.367
YES	42 (7.6%)	26 (9.4%)	
NO	511 (92.4%)	250 (90.6%)	
Acromegalia			0.567
YES	55 (9.9%)	31 (11.2%)	
NO	498 (90.1%)	245 (88.8%)	
Cushing’s syndrome			0.314
YES	38 (6.9%)	14 (5.1%)	
NO	515 (93.1%)	262 (94.9%)	
Thyroid dysfunction			0.824
YES	7 (1.3%)	3 (1.1%)	
NO	546 (98.7%)	273 (98.9%)	
Pituitary apoplexy			0.471
YES	57 (10.3%)	24 (8.7%)	
NO	496 (89.7%)	252 (91.3%)	
Clinical subtype			0.904
Nonfunctional	304 (55.0%)	145 (52.5%)	
PRL secreting	117 (21.2%)	62 (22.5%)	
GH secreting	75 (13.6%)	31 (11.2%)	
ACTH secreting	18 (3.3%)	10 (3.6%)	
TSH secreting	2 (0.4%)	2 (0.7%)	
Plurihormonal	37 (6.7%)	18 (6.5%)	
Tumor size, mm	23.4 ± 5.5	23.7 ± 5.9	0.874
Cavernous sinus invasion			0.206
YES	151 (27.3%)	87 (31.5%)	
NO	402 (72.7%)	189 (68.5%)	
Knosp grading			0.587
0-2	348 (62.9%)	179 (64.9%)	
3-4	205 (37.1%)	97 (35.1%)	
Pseudocapsule-based extracapsular resection			0.817
YES	219 (39.6%)	107 (38.8%)	
NO	334 (60.4%)	169(61.2%)	
Intraoperative CSF leakage			0.574
YES	109 (19.7%)	59 (21.4%)	
NO	444 (80.3%)	217 (78.6%)	
Ki-67≥3			0.882
YES	82 (14.8%)	42 (15.2%)	
NO	471 (85.2%)	234 (84.8%)	
Prolacin (ng/mL)	20.5 ± 26.58	19.5 ± 22.39	0.714
Testosterone (ng/mL)	0.99 ± 1.293	0.99 ± 1.292	0.861
Estradiol (pg/mL)	43.1 ± 41.42	43.7 ± 42.85	0.292
Progesterone (ng/mL)	0.95 ± 2.045	1.15 ± 2.808	0.761
LH (IU/L)	4.61 ± 5.748	5.57 ± 7.377	0.535
FSH (IU/L)	12.2 ± 16.67	12.5 ± 18.75	0.595
TSH (mIU/L)	2.12 ± 2.799	2.35 ± 2.916	0.217
FT3 (pg/mL)	2.58 ± 0.651	2.49 ± 0.609	0.253
FT4 (ng/dL)	1.80 ± 3.183	1.68 ± 3.172	0.193
ACTH (pg/ml)	46.8 ± 25.33	50.3 ± 28.93	0.174
Morning cortisol (μg/dL)	7.48 ± 4.821	6.93 ± 5.297	0.251
Bedtime cortisol (μg/dL)	4.29 ± 2.625	4.03 ± 2.417	0.436
GH (μg/L)	4.23 ± 12.780	3.24 ± 9.498	0.493
IGF-1 (μg/L)	210 ± 244.1	198 ± 225.7	0.751

LH, luteinizing hormone; FSH, follicle-stimulating hormone; TSH, thyroid-stimulating hormone; FT3, free triiodothyronine; FT4, free tetraiodothyronine; ACTH, adrenocorticotropic hormone; GH, growth hormone;IGF-1, insulin-like growth factor-1.

### Analysis of the Risk Factors of PA Recurrence

**Figure 2:**
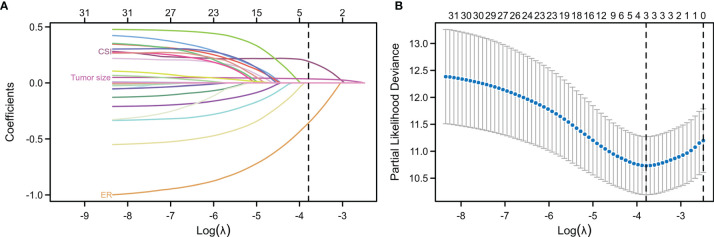
Least absolute shrinkage and selection operator (LASSO) regression analysis for the selection of characteristic parameters. **(A)** Penalty diagram for coefficients of thirty-one characteristic variables. As the penalty coefficient lambda changes, the number of compression variable coefficients increases continuously. Finally, most of the variable coefficients are compressed to zero. **(B)** In the LASSO Logistic regression model, the optimal penalty coefficient lambda was selected by using 10-fold cross-validation and minimization criteria. The optimal lambda (lambda = 3) was selected at the lowest point of the curve, and three variables with non-zero coefficient were selected at the optimal lambda. The characteristic parameters without information were removed to realize automatic selection of characteristic parameters.

**Figure 3:**
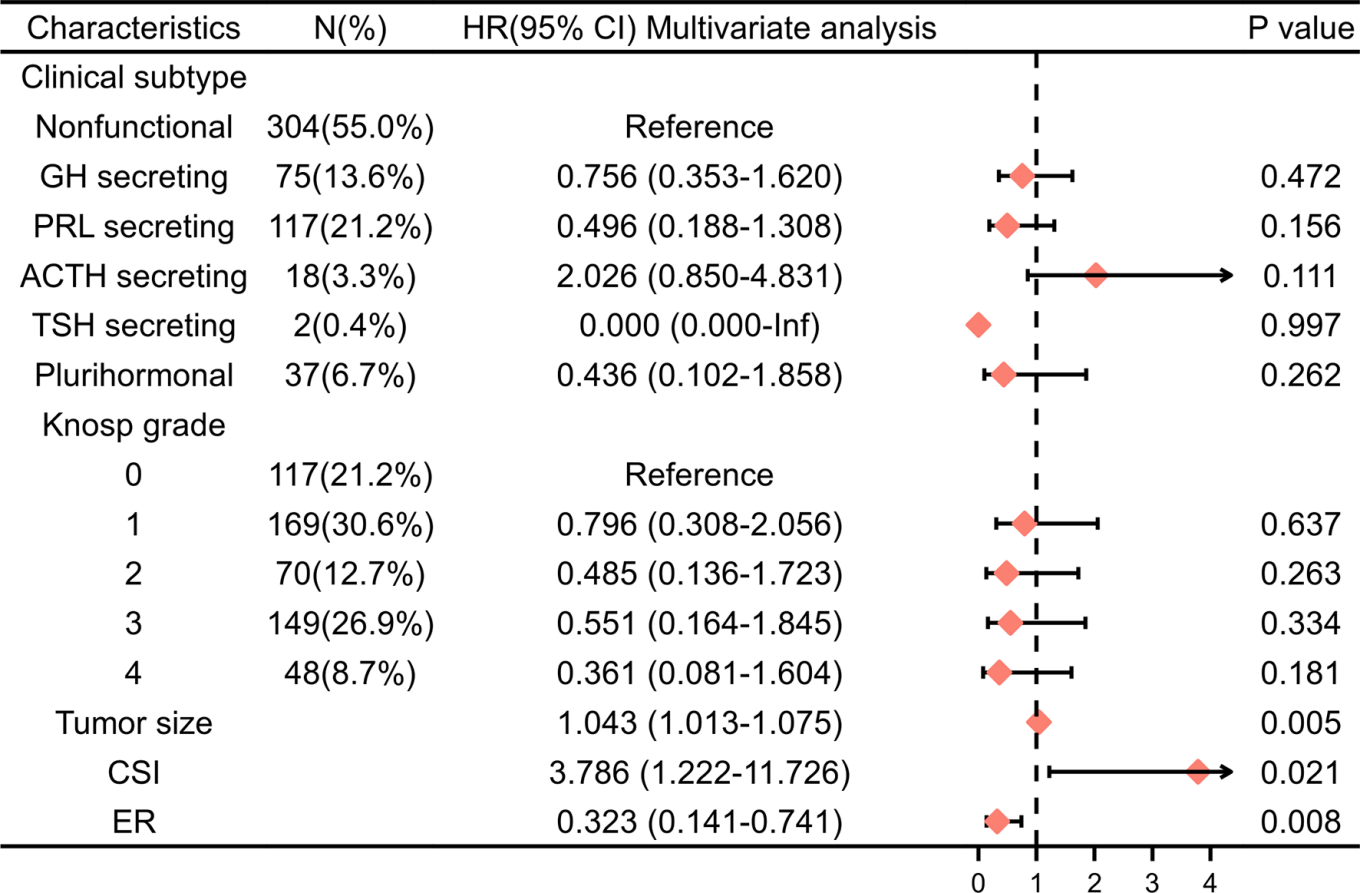
Multivariable Cox regression analysis of PFS in the training cohort. HR, hazard ratio; CI, confidence interval; PRL, prolactin; GH, growth hormone; ACTH, adrenocorticotropic hormone; TSH,thyroid-stimulating hormone; CSI, cavernous sinus invasion; ER, extracapsular resection.

**Figure 4:**
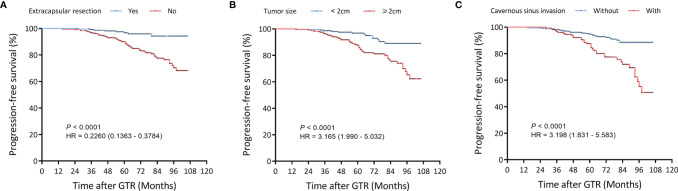
Kaplan–Meier survival curves of filtered risk factors in the training cohort. **(A)** Kaplan–Meier survival curves of tumor size. **(B)** Kaplan–Meier survival curves of pseudocapsule-based extracapsular resection (ER). **(C)** Kaplan–Meier survival curves of cavernous sinus invasion (CSI). GTR, gross-total resection.

Univariate Cox regression and LASSO analysis were used to filter out clinical factors and multivariate Cox regression analysis was applied for further analysis (**Figure 2**, **Table S3**, and **Table S4**). As a result, ER (Hazard ratio [HR], 95% CI: 0.323, 0.141–0.741, P = 0.008), tumor size (HR, 95% CI: 1.043, 1.013–1.075, P = 0.005), and CSI (HR, 95% CI: 3.786, 1.222–11.726, P = 0.021) were incorporated into the multivariate model (**Figure 3**). Corresponding Kaplan–Meier survival curves are shown in **Figure 4**.

### Development of a Nomogram for Postoperative Recurrence

**Figure 5:**
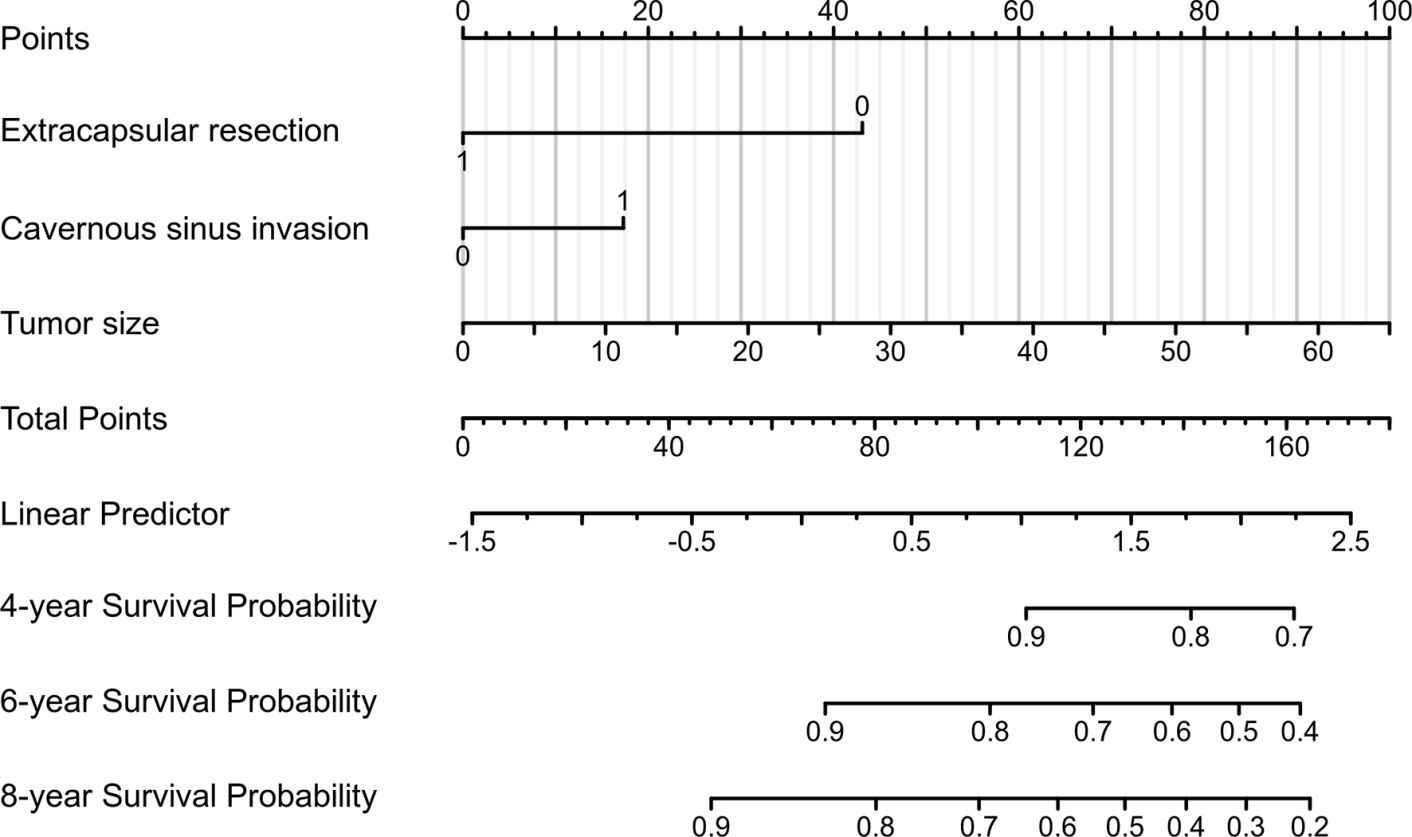
A prognostic nomogram for predicting the 4-, 6-, and 8-year progression-free survival (PFS) probabilities in patients with pituitary adenomas (PAs) after gross-total resection (GTR).

A nomogram was developed to visualise the multivariate model (**Figure 5**). Tumor size was the largest contributor to a prognosis of recurrence, followed by the ER and CSI in the nomogram. To use the nomogram, the respective values were determined using the three factors of an individual patient, and the three values were added to obtain the total value. Subsequently, a line was drawn from the survival axis to determine 4-, 6-, and 8-year PFS probabilities.

### Validation of the Nomogram

**Figure 6:**
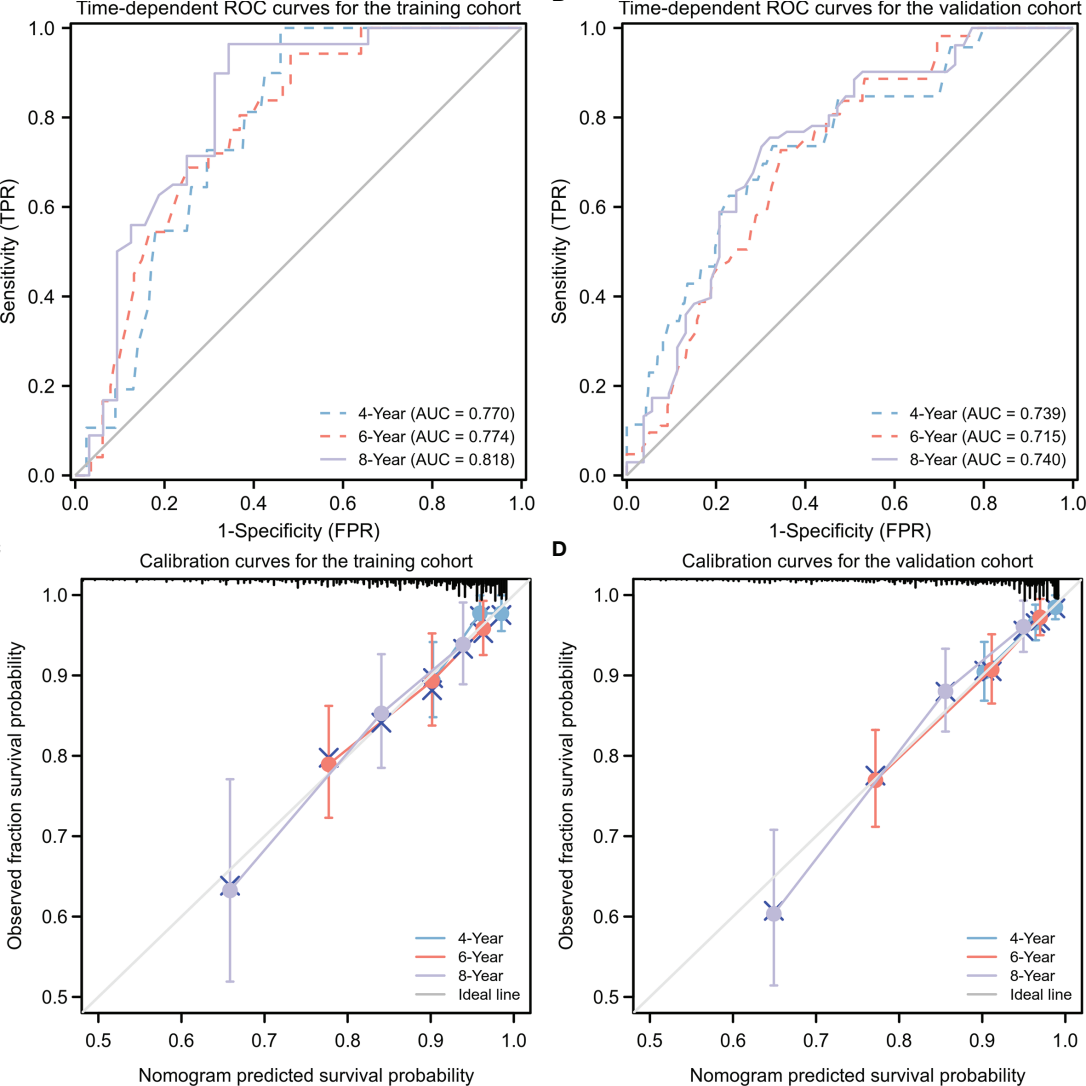
Predictive performance for nomogram model. **(A, B)** Time-dependent ROC analysis of nomogram for 4-, 6-, and 8-year progression-free survival (PFS) probabilities in training cohort **(A)** and validation cohort **(B)**, respectively. **(C, D)** Calibration plots of the nomogram for the training **(C)** and validation **(D)** cohorts at 4, 6, or 8 years. ROC, receiver operating characteristic; TPR, true positive rate; FRR, false positive rate.

The nomogram for PFS prediction showed good predictive capacity with well-fitted calibration curves in both training and validation cohorts (**Figure 6**). The C-indices of the nomogram were 0.776 (95% CI: 0.747–0.806) and 0.714 (95% CI: 0.681–0.747) for the training and validation cohorts, respectively. The AUC of the nomogram was 0.770, 0.774 and 0.818 for 4-, 6-, and 8-year PFS probabilities in the training cohort, respectively; 0.739, 0.715 and 0.740 for 4-, 6-, and 8-year PFS probabilities in the validation cohort, respectively, attesting to the good performance of the nomogram model (**Figure 6**).

### Evaluation of the Clinical Utility of the Nomogram

**Figure 7:**
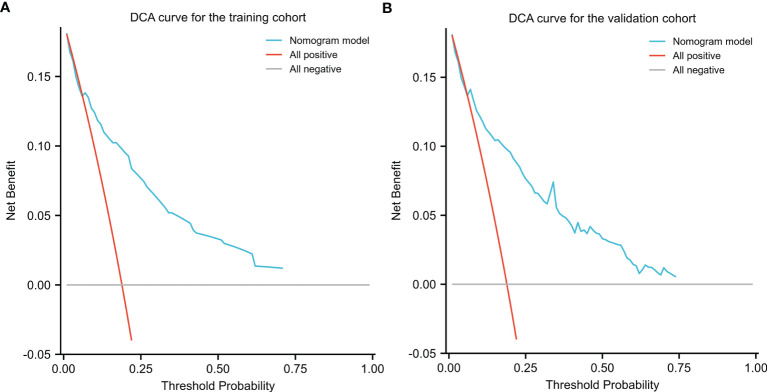
Decision Curve Analysis (DCA) for the nomogram in the training **(A)** cohort and validation cohort **(B)**. Threshold probability and net benefit were represented by the X-axis and the Y-axis, respectively. The blue line represented the net benefit of the nomogram at different threshold probabilities. The area between the “All negative” (gray line) and “All positive” (red line) in the DCA curve indicated the clinical utility of the model.

DCA was applied to evaluate the net benefit of the nomogram for assessing training and validation cohorts, which provided us with insights into the clinical benefit within a reasonable range of threshold probabilities. As a result, DCA revealed that using the nomogram for clinical application likely benefited patients when compared with treating either all or no patients of training and validation cohorts (**Figure 7**).

## Discussion

In the present study, we constructed a clinical nomogram based on pseudocapsule-based ER, tumor size, and CSI for the individualised evaluation of PA recurrence risk after GTR. C-indices, ROC, calibration curves, and DCA showed excellent predictive performance and clinical efficacy when using the nomogram models. Therefore, the nonogram can be used in clinical practice to improve the prediction accuracy of PA recurrence after GTR.

A major new finding from this study is the influence of pseudocapsule-based ER on the risk of PAs recurrence after GTR. The pseudocapsule is the important boundary between pituitary gland and adenoma. Oldfield EH began resecting PAs by dissecting the pseudocapsule from the mid-1980s (16). Kawamata described the important value of excising pseudocapsule when removing growth hormone-secreting tumors to improve rates of endocrine remission and tumor recurrence (11). Pseudocapsule, as a surgical capsule in TTS, may assist the operator in identifying the boundaries of PAs to achieve better resection and remission rates (12, 17). Furthermore, damage to normal pituitary tissue and occurrence of operative complications are reduced (16, 17). Adenoma cells frequently invade and infiltrate the pseudocapsule; as a result, a few tumor cells are easily retained in the pseudocapsule during a conventional intracapsular resection (IR), thus promoting recurrence and preventing the achievement of complete remission (12). Therefore, ER should more completely involve the removal of residual or invasive tumors beyond IR, especially for portions not easily visible. Qu et al. reported that the ER method could be used to achieve higher resection and remission rates than those of IR, a finding consistent with results of prior studies (12, 17–19). Our study demonstrated that ER might reduce risk of PA recurrence after GTR (HR, 95% CI: 0.323, 0.141–0.741, P = 0.008), while careful inspection is essential with the suspicion that tumor cells may remain beyond the main tumor boundary when the pseudocapsule is not fully developed (12).

CSI is a prognostic indicator of the long-term prognosis of PAs (20). Some work has shown that CSI was associated with GTR and recurrence, and the revised Knosp radiological classification was recommended for prediction of surgical outcomes (21–23). GTR rates were negatively correlated with Knosp grade classification, with 56% for grade 3A and only 25% for grade 3B (21). Marta et al. reported that Knosp and revised-Knosp classification showed good diagnostic accuracy in predicting surgical cure (AUC, 0.820), whereas Hardy classification lacked practicality in this purpose (AUC, 0.654) (22), which may indicate that the main prognostic factor was CSI (24). In an 8-year, retrospective, multicenter, cohort study that included 410 patients, Trouillas et al. found that invasiveness was the main prognostic factor for predicting progression-free status (25). Furthermore, a clinicopathologic classification of PAs based on invasion and proliferation was generated to predict relapse/progression-free status (AUC, 0.814) in patients with PAs. They also validated this classification in a prospective single-center cohort comprising 374 postoperative patients followed up for 3.5 years (26).

Although all patients included in this study underwent GTR, complete removal of all adenomas was not guaranteed, especially for PAs with CSI that tended to be associated with invisible or infiltrating adenomas. Zhang et al. indicated that tumor size and CSI were important predictors of GTR in patients with PAs (27). Further, high-field intraoperative MRI facilitated the excision of PAs with CSI, increasing the rate of GTR and decreasing recurrence rates of PAs in endoscopic TSS (27). Thus, GTR improvement leads to improved PFS. CSI was also an independent prognostic indicator of PA recurrence (HR, 95% CI: 3.786, 1.222–11.726, P = 0.021) in our study. Our results suggest that PAs with CSI deserve more attention even after achieving GTR.

The large size of an adenoma may reduce the probability that it is completely resected, which may affect prognosis and recurrence. Hofstetter et al. assessed the effects of adenoma size on resection range, revealing that patients with PAs sized > 10 cm^3^ are most likely to experience residual adenoma (28). GTR was achieved in 90.2% vs 40.0% of adenomas < and > 10 cm^3^, respectively. Further, GTR was complete in 47.6% of a total of 166 patients with adenomas < 3cm versus 9.1% of a total of 77 patients with adenomas > 3 cm (28). Our study also suggested that PAs with larger tumor size are associated with a higher risk of recurrence (HR, 95% CI: 1.043, 1.013–1.075, P = 0.005). Considering its significant impact on the scope and extent of resection, more attention should be paid to the possibility of residual tissue and invasion of large PAs, even when GTR appears to be achieved *via* surgery.

There are several limitations to this study. The single-center study design and absence of external validation are the major limitations, which may introduce the possibility of selection bias and limit the predictive power of the nomogram model. In addition, the study design focuses on ER and PA patients after GTR, which may limit the clinical applicability of the model due to low recurrence rate after GTR and the fact that ER has not been put into use widely enough (1, 12). Despite these limitations, our nomogram was useful for predicting PA recurrence after GTR with good accuracy. Furthermore, pseudocapsule-based ER was introduced as an independent prognostic indicator of PA recurrence after GTR for the first time. This finding deserves more attention and may play a more important role in the treatment and prognosis of PAs. Using the nomogram model, clinicians can communicate more effectively with patients and adjust their treatment plans. Patients with pseudocapsules, for example, could be recommended to undergo pseudocapsule-based ER. Moreover, patients with macroadenomas and CSI should be reminded that they need long-term follow-up, as they are more likely to relapse compared to other patients with PAs, according to our study, even 8 years after GTR.

## Conclusion

This study showed that pseudocapsule-based ER, CSI, and tumor size are independent risk factors for PA recurrence after GTR. Moreover, pseudocapsule-based ER was first introduced as an independent prognostic indicator of PA recurrence after GTR. The nomogram constructed in our study was effective and valuable for predicting PA recurrence after GTR, suggesting its potential utility for assisting neurosurgeons in the development of improved and individualised PA treatment strategies.

## Data Availability Statement

The original contributions presented in the study are included in the article/**Supplementary Material**. Further inquiries can be directed to the corresponding author.

## Ethics Statement

The studies involving human participants were reviewed and approved by the Ethics Committee of Tongji Hospital. Written informed consent for participation was not required for this study in accordance with the national legislation and the institutional requirements.

## Author Contributions

Study design by TL and LL. Data acquisition and analysis by LL, XW, YX and JC. Interpretation of the data by KS and TL. Drafting of the manuscript by LL, XW, YX and JC. Revision of the manuscript by KS and TL. All authors contributed to the article and approved the submitted version.

## Funding

This research was funded by National Natural Science Foundation of China (Grant numbers: 81270865, 82173136) and Transformation and Cultivation Project of Tongji Hospital (Grant number: 2016ZHYX21). The funding institutions had no role in the design of the study, data collection and analysis, the decision to publish, or the preparation of the manuscript.

## Conflict of Interest

The authors declare that the research was conducted in the absence of any commercial or financial relationships that could be construed as a potential conflict of interest.

## Publisher’s Note

All claims expressed in this article are solely those of the authors and do not necessarily represent those of their affiliated organizations, or those of the publisher, the editors and the reviewers. Any product that may be evaluated in this article, or claim that may be made by its manufacturer, is not guaranteed or endorsed by the publisher.

## References

[1] Chen Y Wang CD Su ZP Chen YX Cai L Zhuge QC. Natural History of Postoperative Nonfunctioning Pituitary Adenomas: A Systematic Review and Meta-Analysis. Neuroendocrinology (2012) 96(4):333–42. doi: 10.1159/000339823 22687984

[2] Delgado-López PD Pi-Barrio J Dueñas-Polo MT Pascual-Llorente M Gordón-Bolaños MC. Recurrent Non-Functioning Pituitary Adenomas: A Review on The New Pathological Classification, Management Guidelines and Treatment Options. Clin Trans Oncol (2018) 20(10):1233–45. doi: 10.1007/s12094-018-1868-6 29623588

[3] Day PF Loto MG Glerean M Picasso MFR Lovazzano S Giunta DH. Incidence and Prevalence of Clinically Relevant Pituitary Adenomas: Retrospective Cohort Study in A Health Management Organization in Buenos Aires, Argentina. Arch Endocrinol Metab (2016) 60(6):554–61. doi: 10.1590/2359-3997000000195 PMC10522164 27982201

[4] Daly AF Beckers A. The Epidemiology of Pituitary Adenomas. Endocrinol Metab Clin North Am (2020) 49(3):347–55. doi: 10.1016/j.ecl.2020.04.002 32741475

[5] Aghi MK Chen CC Fleseriu M Newman SA Lucas JW Kuo JS. Congress of Neurological Surgeons Systematic Review and Evidence-Based Guidelines on the Management of Patients With Nonfunctioning Pituitary Adenomas: Executive Summary. Neurosurgery (2016) 79(4):521–3. doi: 10.1227/NEU.0000000000001386 27635956

[6] Buchfelder M Schlaffer SM Zhao Y. The Optimal Surgical Techniques for Pituitary Tumors. Best Pract Res Clin Endocrinol Metab (2019) 33(2):101299. doi: 10.1016/j.beem.2019.101299 31431397

[7] Brochier S Galland F Kujas M Parker F Gaillard S Raftopoulos C. Factors Predicting Relapse of Nonfunctioning Pituitary Macroadenomas After Neurosurgery: A Study of 142 Patients. Eur J Endocrinol (2010) 163(2):193–200. doi: 10.1530/EJE-10-0255 20460423

[8] Greenman Y Cooper O Yaish I Robenshtok E Sagiv N Jonas-Kimchi T. Treatment of Clinically Nonfunctioning Pituitary Adenomas With Dopamine Agonists. Eur J Endocrinol (2016) 175(1):63–72. doi: 10.1530/EJE-16-0206 27150495

[9] Farnoud MR Kujas M Derome P Racadot J Peillon F Li JY. Interactions Between Mormal and Tumoral Tissues at the Boundary of Human Anterior Pituitary Adenomas. An Immunohistochemical Study. Virchows Arch (1994) 424(1):75–82. doi: 10.1007/BF00197396 7981907

[10] Oldfield EH Vortmeyer AO. Development of a Histological Pseudocapsule and Its Use as a Surgical Capsule in the Excision of Pituitary Tumors. J Neurosurg (2006) 104(1):7–19. doi: 10.3171/jns.2006.104.1.7 16509142

[11] Kawamata T Kubo O Hori T. Surgical Removal of Growth Hormone-Secreting Pituitary Adenomas With Intensive Microsurgical Pseudocapsule Resection Results in Complete Remission of Acromegaly. Neurosurg Rev (2005) 28(3):201–8. doi: 10.1007/s10143-005-0384-7 15765245

[12] Lee EJ Ahn JY Noh T Kim SH Kim TS Kim SH. Tumor Tissue Identification in the Pseudocapsule of Pituitary Adenoma: Should the Pseudocapsule be Removed for Total Resection of Pituitary Adenoma? Neurosurgery (2009) 64:(3 Suppl):ons62–9. doi: 10.1227/01.NEU.0000330406.73157.49 19240574

[13] Lyu W Fei X Chen C Tang Y. Nomogram Predictive Model of Post-Operative Recurrence in Non-Functioning Pituitary Adenoma. Gland Surg (2021) 10(2):807–15. doi: 10.21037/gs-21-47 PMC7944052 33708562

[14] Chen Y Cai F Cao J Gao F Lv Y Tang Y. Analysis of Related Factors of Tumor Recurrence or Progression After Transnasal Sphenoidal Surgical Treatment of Large and Giant Pituitary Adenomas and Establish a Nomogram to Predict Tumor Prognosis. Front Endocrinol (2021) 12:793337. doi: 10.3389/fendo.2021.793337 PMC8713699 34970226

[15] Moons KG Altman DG Reitsma JB Ioannidis JP Macaskill P Steyerberg EW. Transparent Reporting of a Multivariable Prediction Model for Individual Prognosis or Diagnosis (TRIPOD): Explanation and Elaboration. Ann Intern Med (2015) 162(1):W1–73. doi: 10.7326/M14-0698 25560730

[16] Taylor DG Jane JA Oldfield EH. Resection of Pituitary Macroadenomas *via* the Pseudocapsule Along the Posterior Tumor Margin: A Cohort Study and Technical Note. J Neurosurg (2018) 128(2):422–8. doi: 10.3171/2017.7.JNS171658 28820308

[17] Jagannathan J Smith R DeVroom HL Vortmeyer AO Stratakis CA Nieman LK. Outcome of Using the Histological Pseudocapsule as a Surgical Capsule in Cushing Disease. J Neurosurg (2009) 111(3):531–9. doi: 10.3171/2008.8.JNS08339 PMC2945523 19267526

[18] Monteith SJ Starke RM Jane JJA Oldfield EH. Use of the Histological Pseudocapsule in Surgery for Cushing Disease: Rapid Postoperative Cortisol Decline Predicting Complete Tumor Resection. J Neurosurg (2012) 116(4):721. doi: 10.3171/2011.12.JNS11886 22283193

[19] Qu X Xu G Qu Y Song T. The Pseudocapsule Surrounding a Pituitary Adenoma and its Clinical Significance. J Neurooncol (2011) 101(2):171–8. doi: 10.1007/s11060-010-0247-0 20526794

[20] Chang EF Zada G Kim S Lamborn KR Quinones-Hinojosa A Tyrrell JB. Long-Term Recurrence and Mortality After Surgery and Adjuvant Radiotherapy for Nonfunctional Pituitary Adenomas. J Neurosurg (2008) 108(4):736–45. doi: 10.3171/JNS/2008/108/4/0736 18377253

[21] Buchy M Lapras V Rabilloud M Vasiljevic A Borson-Chazot F Jouanneau E. Predicting Early Post-Operative Remission in Pituitary Adenomas: Evaluation of the Modified Knosp Classification. Pituitary (2019) 22(5):467–75. doi: 10.1007/s11102-019-00976-6 31286328

[22] Araujo-Castro M Acitores Cancela A Vior C Pascual-Corrales E Rodríguez Berrocal V. Radiological Knosp, Revised-Knosp, and Hardy–Wilson Classifications for the Prediction of Surgical Outcomes in the Endoscopic Endonasal Surgery of Pituitary Adenomas: Study of 228 Cases. Front Oncol (2022) 11:807040. doi: 10.3389/fonc.2021.807040 35127519 PMC8810816

[23] Ouyang T Zhang N Xie S Tang B Li J Xiao L. Outcomes and Complications of Aggressive Resection Strategy for Pituitary Adenomas in Knosp Grade 4 With Transsphenoidal Endoscopy. Front Oncol (2021) 11:693063. doi: 10.3389/fonc.2021.693063 34235083 PMC8255811

[24] Knosp E Steiner E Kitz K Matula C. Pituitary Adenomas With Invasion of the Cavernous Sinus Space: A Magnetic Resonance Imaging Classification Compared With Surgical Findings. Neurosurg (1993) 33:610–8. doi: 10.1227/00006123-199310000-00008 8232800

[25] Trouillas J Roy P Sturm N Dantony E Cortet-Rudelli C Viennet G. A New Prognostic Clinicopathological Classification of Pituitary Adenomas: A Multicentric Case-Control Study of 410 Patients With 8 Years Post-Operative Follow-Up. Acta Neuropathol (2013) 126(1):123–35. doi: 10.1007/s00401-013-1084-y 23400299

[26] Raverot G Dantony E Beauvy J Vasiljevic A Mikolasek S Borson-Chazot F. Risk of Recurrence in Pituitary Neuroendocrine Tumors: A Prospective Study Using a Five-Tiered Classification. J Clin Endocrinol Metab (2017) 102(9):3368–74. doi: 10.1210/jc.2017-00773 28651368

[27] Zhang Z Yang K Xia Y Meng X. High-Field Intraoperative Magnetic Resonance Imaging Increases Extent of Resection and Progression-Free Survival for Nonfunctioning Pituitary Adenomas. World Neurosurg (2019) 127:e925–31. doi: 10.1016/j.wneu.2019.04.001 30974275

[28] Hofstetter CP Nanaszko MJ Mubita LL Tsiouris J Anand VK Schwartz TH. Volumetric Classification of Pituitary Macroadenomas Predicts Outcome and Morbidity Following Endoscopic Endonasal Transsphenoidal Surgery. Pituitary (2012) 15(3):450–63. doi: 10.1007/s11102-011-0350-z 21986872

