# Supplementary material for: Development and Validation of a Prognostic Model for Post-Operative Recurrence of Pituitary Adenomas
Source: Front Oncol. 2022 Apr 28;12:882049. doi: 10.3389/fonc.2022.882049 (PMC9096140; doi:10.3389/fonc.2022.882049)
Supplement: Supplementary file 1 [file Table_1.docx]

**Supplementary Table 1.** Lymphopenia at the beginning and end of RT

|  | **Pre-RT** | **Post-RT** | **p value*** |
| --- | --- | --- | --- |
| Mean PLC (95%CI)-10^9^/L | 1.58 (1.54-1.62) | 0.99 (0.96-1.01) | <0.001 |
| Mean WBC (95% CI)-10^9^/L | 5.69 (5.50-5.88) | 4.38 (4.29-4.48) | <0.001 |
| Lymphopenia (n/%) | 105 (14.3%) | 445 (60.5%) | <0.001 |
| Grade 1 | 84 (11.4%) | 226 (30.7%) |  |
| Grade 2 | 18 (2.5%) | 172 (23.4%) |  |
| Grade 3 | 3 (0.4%) | 45 (6.1%) |  |
| Grade 4 | 0 | 2 (0.3%) |  |

*P values were assessed by matched t-test.
