# Supplementary material for: Development and Validation of a Prognostic Model for Post-Operative Recurrence of Pituitary Adenomas
Source: Front Oncol. 2022 Apr 28;12:882049. doi: 10.3389/fonc.2022.882049 (PMC9096140; doi:10.3389/fonc.2022.882049)
Supplement: Supplementary file 2 [file Table_2.doc]

Supplementary Table 2. Characteristics of patients with PAs in the recurrence and non-recurrence cohorts

| Variable | **Non-recurrence cohort (n=735)** | **Recurrence cohort**  **(n=94)** | P value |
| --- | --- | --- | --- |
| Age,year | 50.6±12.53 | 48.5±12.28 | 0.397 |
| Gender |  |  | 0.517 |
| Female | 323（43.9%） | 38（40.4%） |  |
| Male | 412（56.1%） | 56（59.6%） |  |
| Headache |  |  | 0.365 |
| YES | 187(25.4%) | 28(29.8%) |  |
| NO | 548(74.6%) | 66(70.2%) |  |
| Visual impairment |  |  | 0.456 |
| YES | 369(50.2%) | 51(54.3%) |  |
| NO | 366(49.8%) | 43(45.7%) |  |
| Visual field defect |  |  | 0.445 |
| YES | 16222.0%) | 24(25.5%) |  |
| NO | 573(78.0%) | 70(74.5%) |  |
| Abnormal Menstruation |  |  | 0.777 |
| YES | 61(8.3%) | 7(7.4%) |  |
| NO | 674(91.7%) | 87(92.6%) |  |
| Acromegalia |  |  | 0.419 |
| YES | 74(10.1%) | 12(12.8%) |  |
| NO | 661(89.9%) | 82(87.2%) |  |
| Cushing's syndrome |  |  | 0.392 |
| YES | 48(6.5%) | 4(4.3%) |  |
| NO | 687(93.5%) | 90(95.7%) |  |
| Thyroid dysfunction |  |  | 0.893 |
| YES | 9(1.2%) | 1(1.1%) |  |
| NO | 726(98.8%) | 93(98.9%) |  |
| Pituitary apoplexy |  |  | 0.764 |
| YES | 71(9.7%) | 10(4.3%) |  |
| NO | 664(90.3%) | 84(95.7%) |  |
| Clinical subtype |  |  | **0.013** |
| Nonfunctioning | 385(52.4%) | 64(68.1%) |  |
| PRL secreting | 177(24.1%) | 8(8.5%) |  |
| GH secreting | 94(12.8%) | 12(12.8%) |  |
| ACTH secreting | 25(3.4%) | 5(5.3%) |  |
| TSH secreting | 4(0.5%) | 0(0%) |  |
| Plurihormonal | 50(6.8%) | 5(5.3%) |  |
| Tumor size, mm | 21.8±5.4 | 27.4±6.8 | **<0.001** |
| Cavernous sinus invasion |  |  | **<0.001** |
| YES | 180(24.5%) | 58(61.7%) |  |
| NO | 555(75.5%) | 36(38.3%) |  |
| Knosp grading |  |  | **<0.001** |
| 0-2 | 493(67.1%) | 34(36.2%) |  |
| 3-4 | 242(32.9%) | 60(63.8%) |  |
| Pseudocapsule-based extracapsular resection |  |  | **<0.001** |
| YES | 316(43.0%) | 10(10.6%) |  |
| NO | 419(57.0%) | 84(89.4%) |  |
| Intraoperative CSF leakage |  |  | 0.421 |
| YES | 146(19.9%) | 22(23.4%) |  |
| NO | 589(80.1%) | 72(76.6%) |  |
| Ki-67≥3 |  |  | 0.129 |
| YES | 105(14.3%) | 19(20.2%) |  |
| NO | 630(85.7%) | 75(79.8%) |  |
| Prolacin (ng/mL) | 20.1±24.58 | 22.5±25.39 | 0.341 |
| Testosterone (ng/mL) | 0.92±0.942 | 1.03±1.326 | 0.861 |
| Estradiol (pg/mL) | 46.9±46.881 | 45.0±44.75 | 0.376 |
| Progesterone (ng/mL) | 0.98±1.870 | 0.95±2.011 | 0.643 |
| LH (IU/L) | 5.33±6.720 | 4.57±5.985 | 0.201 |
| FSH (IU/L) | 12.1±15.45 | 12.4±17.23 | 0.849 |
| TSH (mIU/L) | 1.86±1.716 | 2.15±1.916 | 0.254 |
| FT3 (pg/mL) | 2.66±0.521 | 2.51±0.591 | 0.127 |
| FT4 (ng/dL) | 1.90±3.411 | 1.89±3.199 | 0.843 |
| ACTH (pg/ml) | 43.8±25.33 | 48.3±26.40 | 0.107 |
| Morning cortisol (μg/dL) | 8.24±4.649 | 6.96±4.879 | 0.136 |
| Bedtime cortisol (μg/dL) | 4.61±2.909 | 4.01±2.417 | 0.117 |
| GH (μg/L) | 3.23±9.807 | 4.17±10.956 | 0.191 |
| IGF-1 (μg/L) | 173±203.9 | 209±243.4 | 0.339 |

LH, luteinizing hormone; FSH, follicle-stimulating hormone; TSH, thyroid-stimulating hormone; FT3, free triiodothyronine; FT4, free tetraiodothyronine; ACTH, adrenocorticotropic hormone; GH, growth hormone;IGF-1, insulin-like growth factor-1.
