# Supplementary material for: Development and Validation of a Prognostic Model for Post-Operative Recurrence of Pituitary Adenomas
Source: Front Oncol. 2022 Apr 28;12:882049. doi: 10.3389/fonc.2022.882049 (PMC9096140; doi:10.3389/fonc.2022.882049)
Supplement: Supplementary file 3 [file Table_3.doc]

Supplementary Table 3. LASSO analysis in the training cohort

| Characteristics | Coefficient |
| --- | --- |
| Age | 0 |
| Gender | 0 |
| Clinical subtype | 0 |
| KI67 | 0 |
| Cavernous sinus invasion | 0.191605049 |
| Extracapsular resection | -0.357540254 |
| Knosp grade | 0 |
| Intraoperative CSF leakage | 0 |
| Pituitary apoplexy | 0 |
| Tumor size | 0.036145319 |
| Headache | 0 |
| Visual impairment | 0 |
| Visual field defect | 0 |
| Abnormal Menstruation | 0 |
| Acromegalia | 0 |
| Cushing's syndrome | 0 |
| Thyroid dysfunction | 0 |
| Prolacin | 0 |
| Testosterone | 0 |
| Estradiol | 0 |
| Progesterone | 0 |
| LH | 0 |
| FSH | 0 |
| TSH | 0 |
| FT3 | 0 |
| FT4 | 0 |
| ACTH | 0 |
| Morning cortisol | 0 |
| Bedtime cortisol | 0 |
| GH | 0 |
| IGF-1 | 0 |

LH, luteinizing hormone; FSH, follicle-stimulating hormone; TSH, thyroid-stimulating hormone; FT3, free triiodothyronine; FT4, free tetraiodothyronine; ACTH, adrenocorticotropic hormone; GH, growth hormone;IGF-1, insulin-like growth factor-1.
