# Supplementary material for: Development and Validation of a Prognostic Model for Post-Operative Recurrence of Pituitary Adenomas
Source: Front Oncol. 2022 Apr 28;12:882049. doi: 10.3389/fonc.2022.882049 (PMC9096140; doi:10.3389/fonc.2022.882049)
Supplement: Supplementary file 4 [file Table_4.doc]

Supplementary Table 4. Univariate and multivariate analysis of PFS by filtered factors based on the training cohort

| Characteristics | Univariate analysis | | Multivariate analysis | |
| --- | --- | --- | --- | --- |
|  | Hazard ratio (95% CI) | P value | Hazard ratio (95% CI) | P value |
| Clinical subtypes |  |  |  |  |
| Nonfunctional | Reference |  |  |  |
| GH secreting | 0.702 (0.341-1.443) | 0.335 | 0.756 (0.353-1.620) | 0.472 |
| PRL secreting | 0.270 (0.107-0.683) | **0.006** | 0.496 (0.188-1.308) | 0.156 |
| ACTH secreting | 1.632 (0.584-4.559) | 0.350 | 2.026 (0.850-4.831) | 0.111 |
| TSH secreting | 0.000 (0.000-Inf) | 0.996 | 0.000 (0.000-Inf) | 0.997 |
| Plurihormonal | 0.312 (0.075-1.289) | 0.107 | 0.436 (0.102-1.858) | 0.262 |
| Knosp grade |  |  |  |  |
| 0 | Reference |  |  |  |
| 1 | 1.677 (0.701-4.013) | 0.245 | 0.796 (0.308-2.056) | 0.637 |
| 2 | 1.214 (0.364-4.056) | 0.752 | 0.485 (0.136-1.723) | 0.263 |
| 3 | 3.182 (1.438-7.040) | **0.004** | 0.551 (0.164-1.845) | 0.334 |
| 4 | 4.571 (1.832-11.405) | **0.001** | 0.361 (0.081-1.604) | 0.181 |
| Tumor size | 1.055 (1.034-1.077) | **<0.001** | 1.043 (1.013-1.075) | **0.005** |
| Cavernous sinus invasion | 3.112 (1.895-5.111) | **<0.001** | 3.786 (1.222-11.726) | **0.021** |
| Extracapsular resection | 0.221 (0.105-0.465) | **<0.001** | 0.323 (0.141-0.741) | **0.008** |

GH, growth hormone; PRL, prolactin; ACTH, adrenocorticotropic hormone; TSH, thyroid-stimulating hormone.
